# Three-dimensional imaging of a peripilar cast and compound follicle in frontal fibrosing alopecia

**DOI:** 10.1016/j.jdcr.2022.02.036

**Published:** 2022-03-11

**Authors:** Curtis T. Thompson, Maria Abril Martinez Velasco, Antonella Tosti

**Affiliations:** a CTA Pathology, Portland, Oregon; b Departments of Dermatology and Pathology, Oregon Health and Sciences University, Portland, Oregon; c Hospital Médica Sur, Mexico City, Mexico; d Department of Dermatology, University of Miami Miller School of Medicine, Miami, Florida

**Keywords:** acanthosis, alopecia, cicatricial alopecia, compound follicle, dermatoscopy, follicular hyperkeratosis, frontal fibrosing alopecia, interface dermatitis, lichen planopilaris, lichen planus, lymphocytic, pathology, peripilar cast, reflectance confocal microscopy, transverse sections, trichoscopy, FFA, frontal fibrosing alopecia, RCM, reflectance confocal microscopy

## Introduction

Frontal fibrosing alopecia (FFA) has become a frequent diagnosis for dermatologists and dermatopathologists. Even though the disease can be recognized clinically, pathologic confirmation is often necessary, particularly in early disease. We have previously shown that a 2-mm dermatoscopy-guided biopsy is effective in diagnosing the disease, thereby allowing histopathologic confirmation, despite the presence of minimal scarring.^1^ The aim of this study was to better characterize a single disease focus in FFA, namely the peripilar cast, with a variety of imaging modalities. On the skin surface, dermatoscopy and in vivo reflectance confocal microscopy (RCM) were used to visualize the changes occurring at the skin surface and in the follicular ostium. Histopathology was used to characterize the disease focus not only at the skin surface but also in the subjacent dermis and subcutis. Using the images obtained from the 3 different imaging modalities, we constructed a 3-dimensional diagram of this single disease focus.

## Case report

A patient with a complaint of recession of the frontal hairline was clinically evaluated, and a focus of active disease was dermatoscopically identified and marked. The site was confirmed after marking again using the dermatoscope. The disease focus had a tuft of 2 hairs, which were surrounded by a dilated follicular ostium and a peripilar cast*.* RCM was then performed using a Vivascope 3000 imaging system (Caliber Imaging and Diagnostics). The Vivascope 3000 imaging system is a handheld reflectance confocal microscope that provides high resolution images of the skin surface, including the superficial portion of adnexal structures. The RCM imaging provided an en face 750-μm^2^ field of view, with a resolution of approximately 1 μm and an optical sectioning of approximately 3 μm. A 2-mm punch biopsy specimen was then obtained from the site and processed in the manner previously described.^1^ In this method, the 2-mm biopsy specimen is not bisected, instead it is sectioned transversely (horizontally) beginning at the epidermal surface and continuing through the entirety of the tissue segment. Finally, a 3-dimensional sketch of the disease focus was created by a professional medical artist using evidence from the dermatoscopic images, the RCM images, and the collection of transverse hematoxylin-eosin–stained sections. The accuracy of the 3-dimensional sketch was confirmed by the study authors.

**Fig 1:**
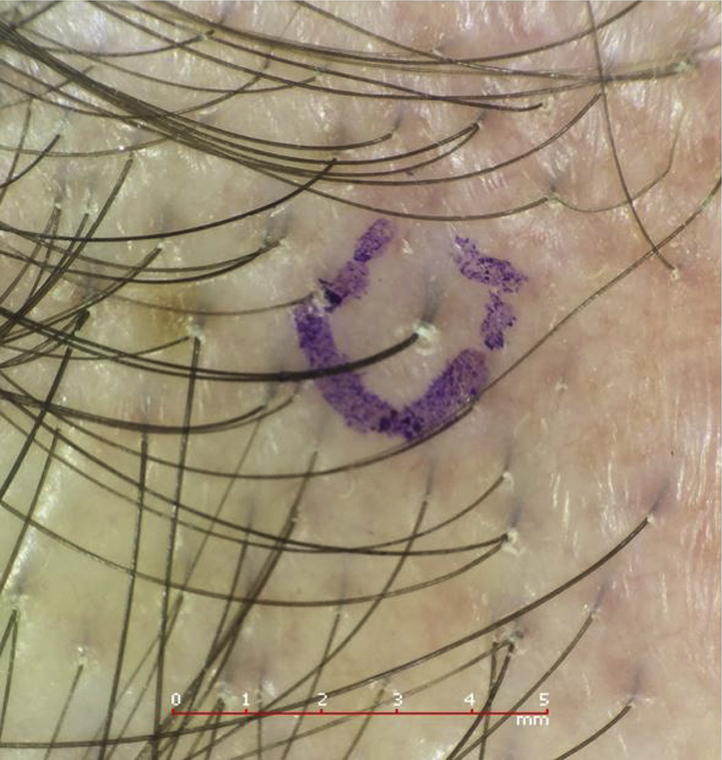
Peripilar cast. Dermatoscopic imaging of 2 “lonely” hairs with a peripilar cast, marked in purple ink, for in vivo reflectance confocal microscopy imaging and subsequent biopsy.

**Fig 2:**
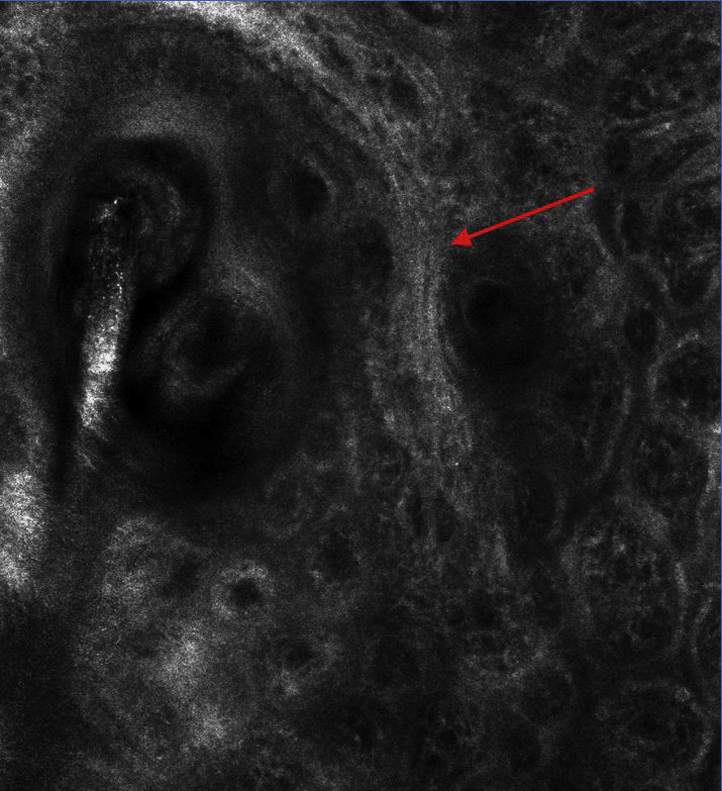
In vivo reflectance confocal microscopy imaging of the peripilar case. Two follicles in the center of a dilated ostium with a hyperkeratotic, acanthotic epithelial rim (*arrow*) were identified. Of note, only 1 of the 2 hair shafts was out of focus and not well visualized, even though there were 2 follicles present. In the base of the widened follicular ostium, hyperkeratosis and 2 fused follicles were observed, representing a compound follicle (Vivascope 3000 system, 750 μm^2^ field of view with 1 μm resolution and 3 μm optical sectioning).

**Fig 3:**
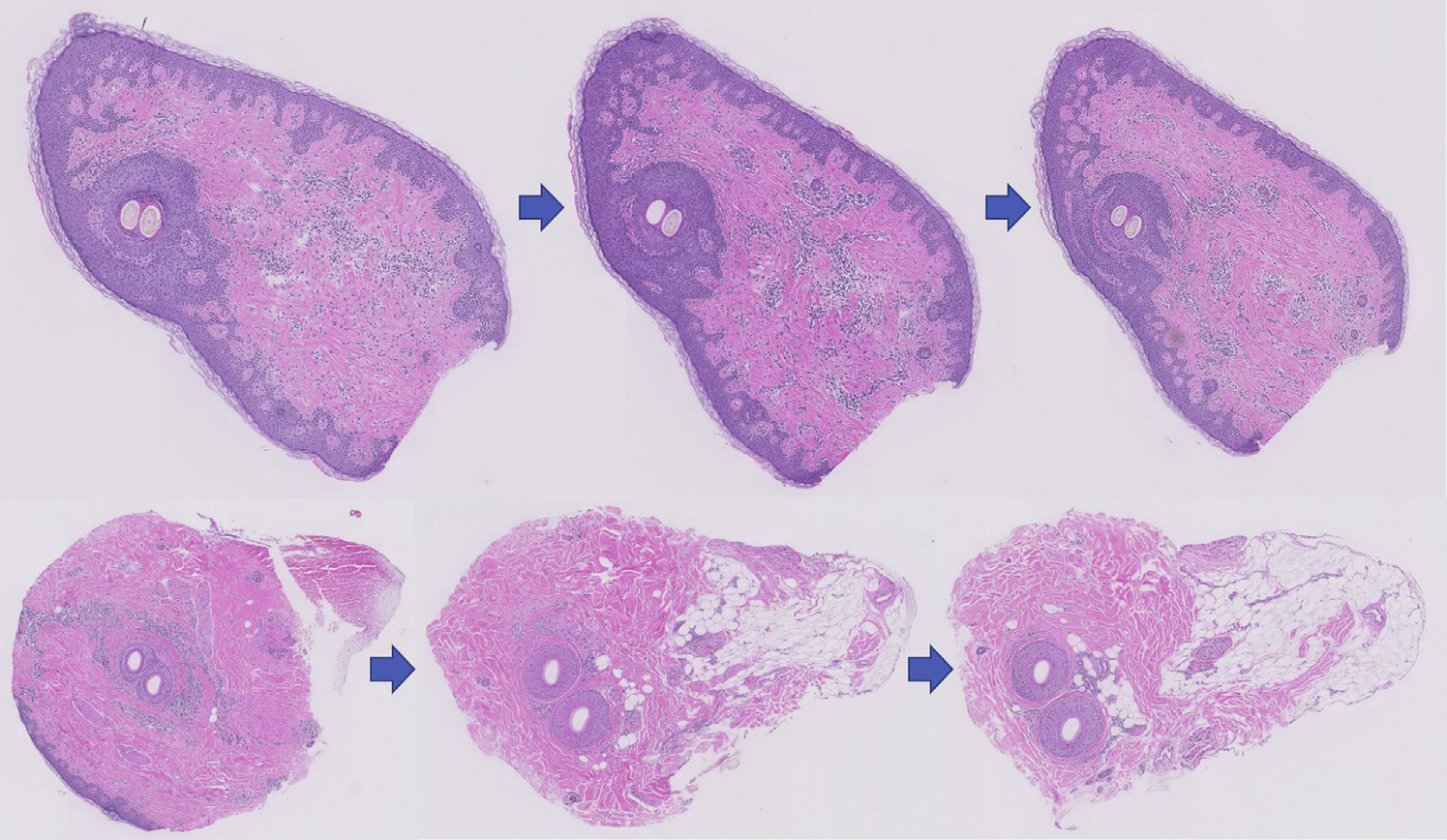
Disease focus. Transverse (horizontal) sections of a 2-mm punch biopsy specimen demonstrating sampling through the entire tissue segment (Hematoxylin-eosin stain; original magnification: ×200). Starting at the skin surface (*upper left*), 2 hair shafts were seen in the follicular infundibulum. Progressively deeper sections (*left to right*) through the dermis of the biopsy specimen showed a compound follicle with 2 hair shafts, gray-staining perifollicular fibrosis, and a surrounding perifollicular infiltrate of lymphocytes. The deepest sections (*lower right*) near the dermal-subcutaneous junction showed near-normal appearing lower root segments of the 2 hair follicles. a feature typical of FFA. (Hematoxylin-eosin stain; original magnification: ×400).

**Fig 4:**
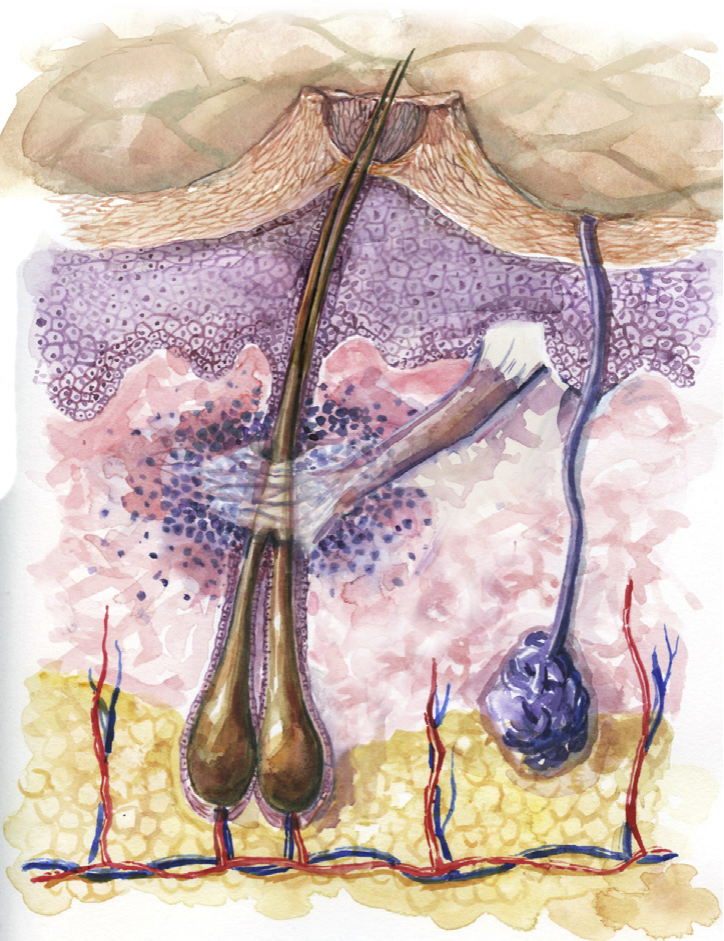
Frontal fibrosing alopecia. A 3-dimensional sketch of the same disease focus of FFA constructed using the dermatoscopic, in vivo confocal and transverse (horizontal) histopathologic sections. The 2 follicles exit from the dilated follicular ostium with its hyperkeratotic rim, which produces the visible volcano-like peripilar cast. The 2 follicles fuse at the level of the isthmus and superficial infundibulum, where there is perifollicular fibrosis and lymphocytes. The lower root segment of the 2 follicles appears near normal.

The 3 imaging modalities—dermatoscopy, RCM, and histopathologic analysis—have jointly characterized a disease focus of FFA as seen in Fig 1. Dermatoscopy identified a tuft of 2 hairs, a peripilar cast, and a compound follicle. RCM showed a dilated follicular opening with a rim of acanthotic, hyperkeratotic epithelium surrounding the follicular ostium. Only 1 of the 2 hair shafts was seen in the RCM image, but the infundibulum of the compound follicle was evident, as seen in Fig 2. On analysis of the hematoxylin-eosin–stained transverse (horizontal) section, the initial sections at the skin surface correlated with the dermatoscopic and RCM images. The infundibular follicular epithelium was widened with 2 hair shafts, thereby explaining the widened follicular opening forming the peripilar cast, as seen in the upper left portion of Fig 3. A slightly deeper section showed the compound follicle with 2 hair shafts, gray-staining perifollicular fibrosis, and a surrounding perifollicular infiltrate of lymphocytes, as seen in the upper right section of Fig 3. The deep sections of the lower reticular dermis and subcutis showed the lower root segments of the 2 hair follicles without evident abnormalities, as seen in the lower right portion of Fig 3. All of these findings are represented in the 3-dimensional sketch created by a professional medical artist, as seen in Fig 4.

## Discussion

By using multiple imaging modalities of a single focus of active disease in FFA, we have been able to provide a precise 3-dimensional description of the dermatoscopically-identified peripilar cast. The collection from the different imaging modalities shows that the peripilar cast is the result of a rim of acanthotic, hyperkeratotic epithelium surrounding the follicular ostium. This volcano-shaped structure overlies a subjacent compound follicle where there is an active lymphocyte-predominant interface dermatitis. The peripilar epidermal acanthosis associated with FFA in the follicular epithelium is analogous to the acanthosis seen in the interadnexal epidermis in lichen planus. Our 3-dimensional imaging precisely showed that the disease is confined to the infundibulum and superficial isthmus, where 2 follicles have fused to form a compound follicle.

To our knowledge, this is the first study to characterize the peripilar cast and is medically significant because it shows an example of the correlation between a clinically apparent feature of a disease and its corresponding histopathologic finding. The study also emphasizes the need for clinicians to carefully identify and sample an active disease focus in FFA in order to make a definitive diagnosis.

## Conflicts of Interest

Dr Tosti is a consultant for DS Laboratories, Monat Global, Almirall, Thirty Madison, Lilly, LEO Pharmaceuticals, Bristol Myers Squibb, and P&G. Drs Thompson and Velasco have no conflicts of interest to declare.
